# Advancements in reperfusion rates and quality of care for ST-segment elevation myocardial infarction: a ten-year evaluation of Salvador's STEMI network

**DOI:** 10.3389/fcvm.2024.1381504

**Published:** 2024-07-22

**Authors:** Pollianna Souza Roriz, Isabella Bonifácio Brige Ferreira, Fabiana Benevides Pontes, Antônio Machado, Tércio Caires Aguiar, Marcos Antônio Almeida Matos, Ivan Mattos Paiva Filho, Rodrigo Carvalho Menezes, Bruno Bezerril Andrade

**Affiliations:** ^1^ Departamento de Cardiologia, Serviço de Atendimento Móvel de Urgência (SAMU), Salvador, Brazil; ^2^ Departamento de Estimulação Cardíaca Artificial, Hospital Ana Nery, Salvador, Brazil; ^3^ Pós Graduação em Medicina e Saúde Humana, Escola Bahiana de Medicina e Saúde Pública (EBMSP), Salvador, Brazil; ^4^ Curso de Medicina, Universidade Salvador (UNIFACS), Salvador, Brazil; ^5^ Departamento de Atenção às Urgências, Secretaria Municipal de Saúde, Salvador, Brazil; ^6^ Instituto de Pesquisa Clínica e Translacional, Curso de Medicina, Faculdade ZARNS, Salvador, Brazil; ^7^ Laboratório de Pesquisa Clínica e Translacional, Instituto Gonçalo Moniz, Fundação Oswaldo Cruz (FIOCRUZ), Salvador, Brazil

**Keywords:** ST-elevation myocardial infarction, reperfusion, universal health care, developing countries, quality improvement

## Abstract

**Background:**

Continuous investment and systematic evaluation of program accomplishments are required to achieve excellence in ST-segment elevation myocardial infarction (STEMI) care, especially in resource-limited settings. Therefore, this study evaluates the impact of problem-driven interventions on reperfusion use rate in a long-term operating STEMI network from a low- to middle-income country.

**Methods:**

This is a healthcare improvement evaluation study of Salvador's public STEMI network in a quasi-experimental design, comparing data from 2009 to 2010 (pre-intervention) and 2019-2020 (post-intervention). There were evaluated all confirmed STEMI cases assisted in both periods. The interventions, implemented since 2017, included: expanding the support team, defining criteria to be a spoke, and initiating continuous education activities. The primary outcome was the rate of patients undergoing reperfusion, with secondary outcomes being time from door-to-ECG (D2E) and ECG-to-STEMI-team trigger (E2T).

**Results:**

Over ten years, the network's coverage increased by 300,000 individuals, and expanded by 1,800 km^2^. A total of 885 records were analyzed, 287 in the pre-intervention group (182 men [63·4%]; mean [SD] age 62·1 [12·5] years) and 598 in the post-intervention group (356 men [59·5%]; mean [SD] age 61.9 [11·8] years). It was noticed a substantial increase in reperfusion delivery rate (90 [31%] vs. 431 [73%]; *P *= 001) and reductions in time from D2E (159 [83–340] vs. 29 [15–63], *P *= 001), and E2T (31 [21–44] vs. 16 [6–40], *P *= 001).

**Conclusion:**

The strategies adopted by Salvador's STEMI network were associated with significant improvements in the rate of patients undergoing reperfusion and in D2E and E2T. However, the mortality rate remains high.

## 1 Introduction

Despite significant advancements in the treatment of ST-Segment Elevated Myocardial Infarction (STEMI), it remains a significant global health concern. In 2021, ischemic heart disease, which includes STEMI, was responsible for 9.4 million deaths worldwide (95% Uncertainty Interval: 8.8–9.9 million) (1). Regional STEMI networks, which integrate various healthcare facilities, have been particularly effective in ensuring timely reperfusion and improving patient outcomes (2–6). However, in low- and middle-income countries (LMICs), these networks often face considerable challenges that compromises their performance (7).

Salvador, the fifth most populous city in Brazil, pioneered the implementation of a STEMI care network in 2009. This initiative aimed to optimize care by linking several public emergency units with Percutaneous Coronary Intervention (PCI)-Capable Centers, thereby forming a cohesive team to organize STEMI care in the city. A comprehensive study published in 2012 documented its implementation, highlighting the epidemiological context, the network's care coordination model, and encountered barriers (8).

The 2012 study identified several key challenges that significantly hindered the growth and effectiveness of the network. Infrastructure limitations included inadequate health system informatization, insufficient hospital beds in PCI centers, and capacity discrepancies across health facilities. The network's expansion outpaced infrastructure growth, leading to an increasing insufficiency in response capacity. Additionally, professional training and public health education on Acute Coronary Symptoms identification and management were lacking. Systemic management and coordination issues included STEMI care times that exceeded recommended guidelines, lack of standardized interinstitutional protocols, limited information sharing, and response times frequently surpassing 60 s.

In response to these challenges, several targeted interventions were implemented to improve the network's performance. This study evaluates the impact of these strategic interventions on reperfusion rates within Salvador's STEMI network over a ten-year period. By comparing data from the pre-intervention and post-intervention periods, improvements in quality-of-care outcomes, structural changes, and persistent challenges are assessed, presenting valuable insights into the long-term performance of a STEMI network in a LMIC.

## 2 Methods

### 2.1 Study design

This is a healthcare improvement evaluation study of Salvador's STEMI network, employing a quasi-experimental design. Data from two periods, pre-intervention (2009–2010) and post-intervention (2019–2020), were compared, adhering to the Standards for Quality Improvement Reporting Excellence (SQUIRE) guidelines (9).

### 2.2 Context

**Figure 1:**
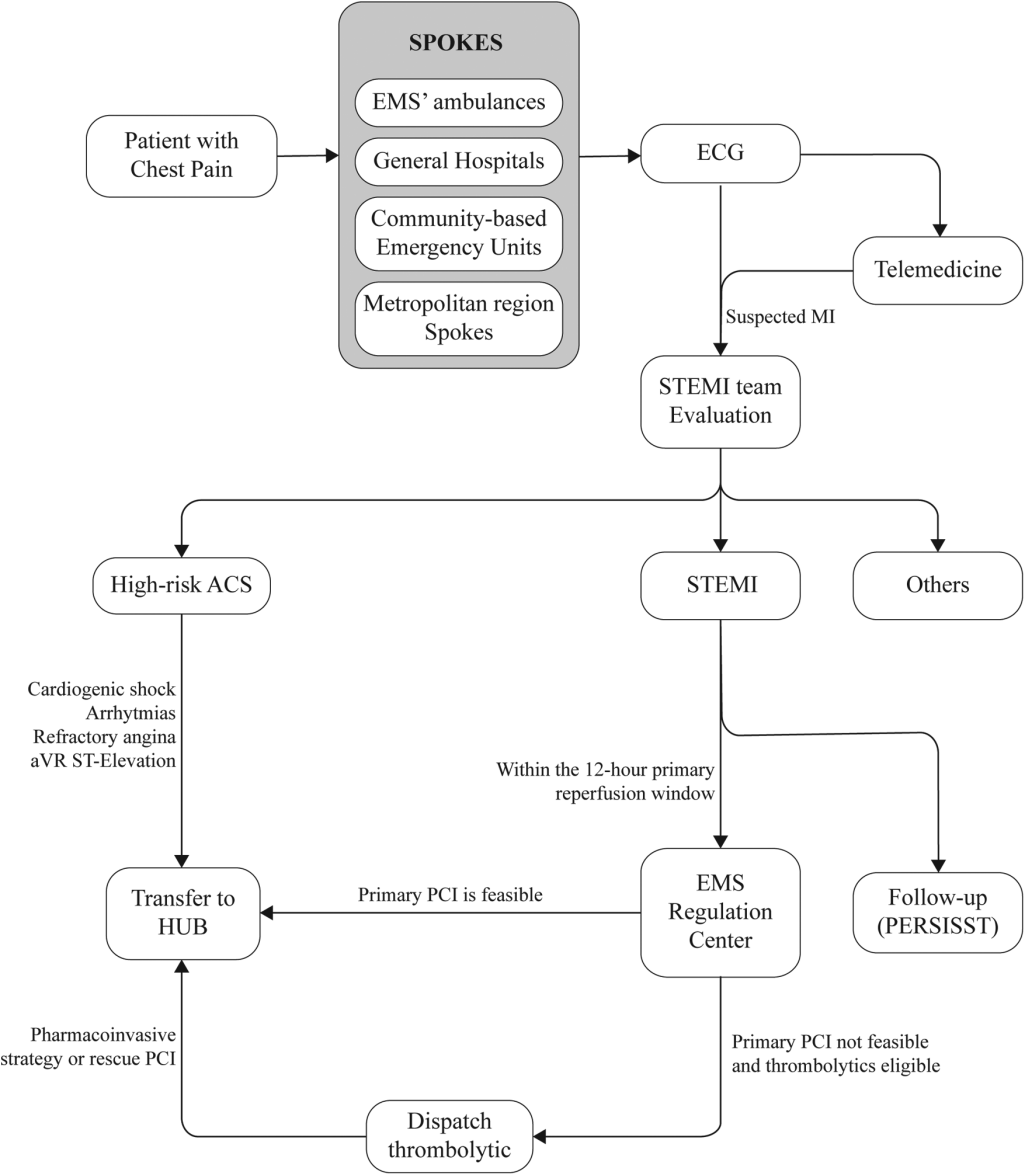
Flowchart of operation of salvador's STEMI network. STEMI indicates ST-segment–elevation myocardial infarction; ACS, acute coronary syndrome; ECG, electrocardiogram; EMS, emergency medical service; PCI, percutaneous coronary intervention; and PERSISST, Pesquisa Soteropolitana de Infarto Agudo do Miocárdio com Supradesnivelamento do segment ST.

Salvador's STEMI Network operates with funding from the Brazilian public health system. It is coordinated by the local Emergency Medical Service (EMS) in collaboration with Salvador's Health Departments, adopting a hub-and-spoke model. The EMS comprises various first medical contact (FMC) points, termed spokes, such as EMS ambulances, community-based emergency units and general hospitals. Additionally, there are PCI-Capable Centers, referred to as hubs, offering 24/7 support for primary PCI, as depicted in Figure 1 (10, 11).

The primary pathway for network activation involves healthcare professionals at the spokes identifying suspected STEMI patients and connecting with the STEMI-network's team through an encrypted instant messaging application available on all spoke computers. This application is used to share patient information and electrocardiogram (ECG) data. An alternative pathway involves a partnership with a telemedicine company, which remotely interprets ECGs from specific community-based emergency units. When a STEMI-compatible ECG is identified, the company triggers the network's team through the instant messaging application, providing patient's name and the location of the unit (Figure 1).

In both scenarios, on-call trained and supervised medical students contact the spoke to collect patient data according to a pre-established form. Cases are then discussed with an on-call cardiologist. If a suspected STEMI diagnosis is confirmed within 12 h of symptom onset, the team assesses resource availability, adhering to national and international STEMI guidelines (12, 13). Based on these factors, the optimal reperfusion strategy is determined, and the EMS regulation center is then promptly activated, dispatching an advanced ambulance equipped with a nurse, physician and driver. This ambulance either rushes the patient to a hub for immediate admission to the catheterization laboratory or delivers fibrinolytic medication to the healthcare unit for local administration.

### 2.3 Interventions

To address the challenges highlighted in the previous study, which impeded network growth and effectiveness, the following problem-driven interventions were implemented:

a) Team composition: In 2009, the program consisted of seven medical students supervised by one cardiologist affiliated with the EMS. Each student received a scholarship and was responsible for occurrences one day per week, while the cardiologist was on duty 24 h a day, seven days per week. By 2017, the STEMI team expanded significantly to 26 members, including 15 medical students, five nursing students, and six cardiologists now affiliated with the network. The stipend paid to students increased by 2.5 times. Medical students, now working in pairs on 12-hour shifts, are assigned to respond to spoke calls within 60 s to collect patient data, discuss the case with an on-call cardiologist, and to contact the hub to determine bed availability. Cardiologists took turns working 12-hour shifts, allowing a more sustainable workload and ensuring availability. Nursing students took on important roles in peri-STEMI follow-up, documenting data on complications, clinical management and discharge plans, and managing of a detailed database that serves as a source of key performance indicators and informs essential public and legal measures. The team provided specialized support 24/7.
b) Criteria for spoke designation: Healthcare facilities aiming for “spoke” status had to meet minimum criteria for STEMI management. They must guarantee: (i) availability of first-line medications for STEMI; (ii) at least one portable ECG machine; (iii) on-site physicians; (iv) commitment to participate in regular training sessions; and (v) willingness to share decisions and data with the STEMI-network. Additionally, the availability of fibrinolytic therapy was encouraged by not mandatory, as only a few EMS ambulances and general hospitals were equipped to provide it.
c) Continuous education and facility visits: Since 2017, the network has implemented a program of continuous education semiannually for universities and quarterly for spokes. These activities aimed at training students and emergency healthcare professionals from various disciplines in identifying suspected cases of STEMI and the proper procedures for activating the STEMI network. Additionally, regular visits to all facilities within the network were implemented to distribute and reinforce standardized protocols for STEMI care through printed flowcharts. Moreover, these visits also served to ensure the proper functioning of the communication application, to assess the compliance with the criteria for spoke designation, and to maintain a point of contact with each spoke, with clearly defined roles and leadership. This structure ensures that each facility has a dedicated team member responsible for overseeing STEMI protocols and coordinating with the network, thereby enhancing overall efficiency and responsiveness.

#### 2.3.1 Data collection

**Figure 2:**
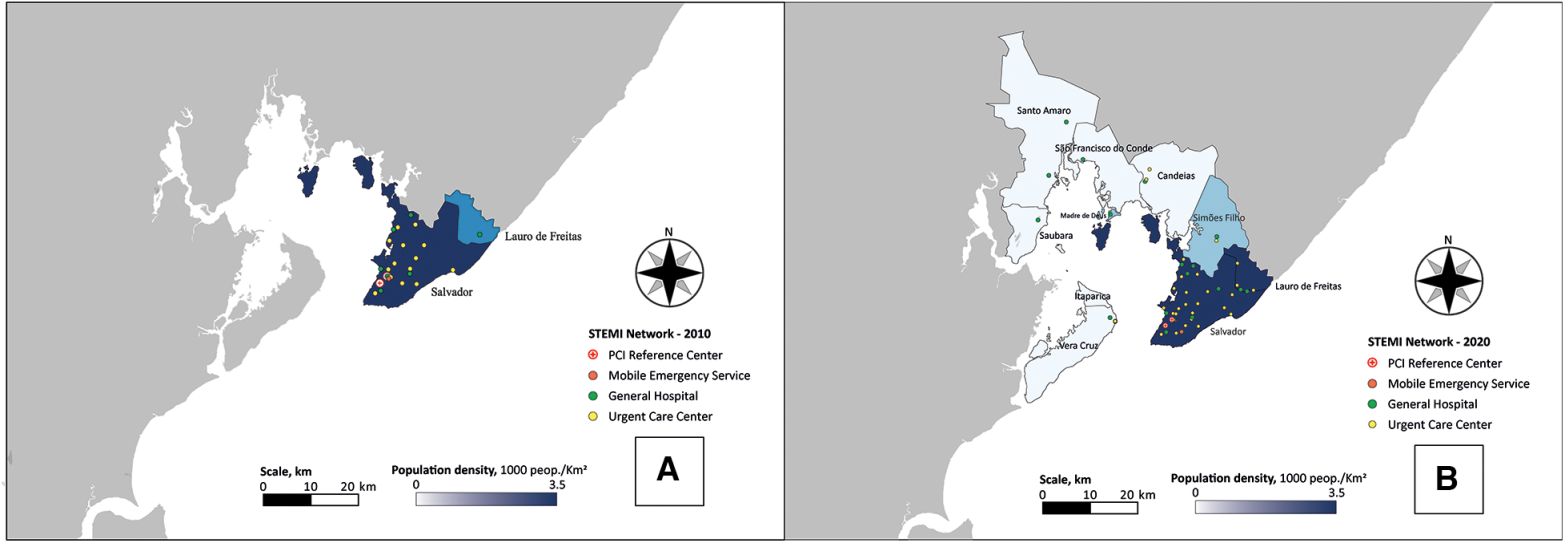
Geographic distribution of public healthcare facilities part of the integrated regional STEMI network of salvador, Bahia, Brazil and its metropolitan area through the decade. (**A**) Units by the time of the first publication, in 2012 (total coverage area: 751.84km^2^). (**B**) Health units currently covered by the STEMI network (2021), highlighting its expansion (total coverage area: 2,553.40km^2^).

Data from 2009 to 2010 were sourced from the study by Solla et al. (8) Data from 2019 to 2020 were prospectively collected from service records maintained by the team STEMI network team. The collected data included patients demographics (age, sex), risk factors, clinical presentation, GRACE Score, various time metrics in STEMI management (e.g., door-to-ECG [D2E] and ECG-to-STEMI team trigger [E2T]), and in-hospital mortality. All suspected STEMI cases assisted by the network during the study period were included, with non-confirmed STEMI cases being excluded (Figure 2). The primary outcome was the rate of patients undergoing reperfusion. Secondary outcomes included time from door-to-ECG and time from ECG-to-STEMI team trigger.

### 2.4 Statistical analysis

Normality was assessed using the Shapiro-Wilk test. Descriptive statistics were employed to summarize the data. Continuous non-normal variables were reported as medians and interquartile ranges (IQR), while continuous normal variables were reported as means and standard deviations (SD). Categorical variables were reported as absolutes number and percentages. Fisher's Exact test was used for comparing proportions, the one-sample *T*-test for comparing means, and the one-sample Wilcoxon's signed-rank test for comparing medians. Geospatial information was incorporated into the study using public data from the Brazilian Institute of Geography and Statistics (IBGE) and analyzed using QGIS (QGIS Geographic Information System, version 3.38 QGIS Association) (14, 15).

## 3 Results

### 3.1 STEMI network in 2019–2020

**Figure 3:**
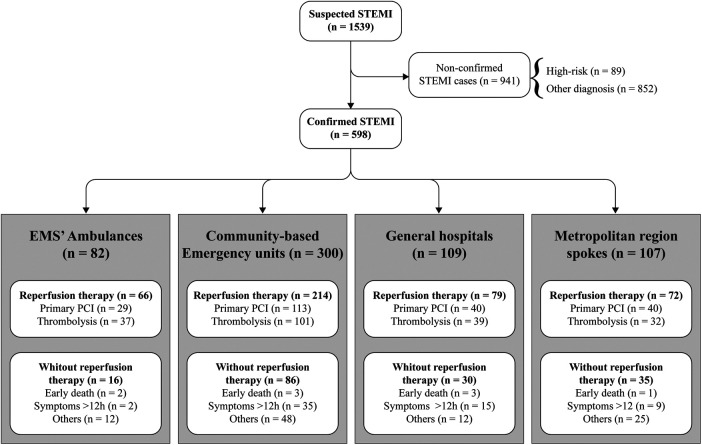
Point of entrance of the cases attended by the STEMI network and the reperfusion strategy performed. STEMI indicates ST-segment–elevation myocardial infarction; EMS, emergency medical service and PCI, percutaneous coronary intervention.

The coverage area of Salvador's STEMI network has experienced substantial expansion, increasing from 751 km^2^ to an extensive 2,586 km^2^ (Figure 3). This expansion includes the integration of eight new municipalities surrounding Salvador, with Human Development Indexes (HDI) ranging from 0.617 to 0.759. Consequently, the program now extends its support to approximately 2.9 million individuals, compared to 2.7 million reported in 2010. Between 2010 and 2020 the number of spokes expanded from 21 to 46, leading to a range of distances between the spokes and hubs ranging from 1.00 km to 103.00 km. Remarkably, the number of hubs remained unchanged over the past decade.

### 3.2 Outcome measures, and quality improvement in STEMI care

A total of 885 records were analyzed, being 287 in the pre-intervention group and 598 in the post-intervention group. The mean age of patients was 62.1 years (SD: 12.5) in the pre-intervention group and 61.9 years (SD: 11.8) in the post-intervention group. The pre-intervention group had a higher prevalence of inferior wall STEMI cases and a greater number of individuals with a history of prior myocardial infarction. The baseline characteristics for both periods are presented in Table 1.

**Table 1:** Baseline characteristics of the 885 STEMI cases assisted.

Characteristics	2019–2020	2009–2010	*p*-value	Missing data^a^
	(*n* = 598)	(*n* = 287)		
Age, mean (SD), years	61.9 ± 11.8	62.1 ± 12.5	0.628	–
Males	356 (59.5)	182 (63.4)	0.586	–
Risk factors				
Hypertension	416 (69.6)	193 (73.7)	0.764	–
Diabetes	204 (34.1)	97 (37.5)	0.999	–
Dyslipidemia	60 (10.0)	^b^	–	–
Prior myocardial infarction	60 (10.0)	51 (21.5)	0.004	–
Features at presentation				
Blood pressure, mean (SD), mmHg				
Systolic	148 (36)	^b^	–	9 (1.5)
Diastolic	89 (22)	^b^	–	9 (1.5)
Heart rate, mean (SD), bpm	85 ± 20	77.9 ± 17.7	<0.001	8 (1.3)
Killip				9 (1.5)
I	529 (88.4)	^b^	–	
II	38 (6.3)	^b^	–	
III	10 (1.7)	^b^	–	
IV	12 (2.0)	^b^	–	
Location of infarct				2 (0.3)
Anterior	321 (53.7)	218 (56.8)	0.346	
Inferior	238 (39.8)	137 (47.7)	<0.001	
Other	37 (6.2)	^b^	–	
Grace score (points)	109 ± 27	^b^	–	109 (18.2)
Low risk	253 (42.3)	^b^	–	
Intermediate risk	171 (28.6)	^b^	–	
High risk	65 (10.9)	^b^	–	
First medical contact <12 h since symptom onset	488 (81.6)	138 (48.0)	<0.001	82 (13.7)
Completion of ECG <12 h since symptom onset	523 (87.5)	119 (41.4)	<0.001	39 (6.5)
In-hospital death	90 (15)	^b^	–	22 (3.6)

Data are presented as the number (percentage) of participants unless otherwise indicated.

^a^ Missing data is referent for 2019–2020 cohort.

^b^ Blank cells represent data not available between 2009 and 2010. Electrocardiogram (ECG), Beats per minute (bpm), millimeters of mercury (mmHg), Not applicable (–).

Community-based Emergency units were the main point of first medical contact in both periods (152 [52.9%] vs. 300 [50.1%], *p*-value = 0.470). However, there was an increase in the number of calls by ambulances (3 [1.0%] vs. 82 [13.7%], *p*-value = 0.001) (Figure 2). Although approximately half of the patients still took around two hours to seek care, comparing the study periods revealed a reduction in the median time from pain-to-first medical contact (P2FMC) (180 min [IQR: 90–473] to 122 min [IQR: 50–265], *p*-value = 0.003). Furthermore, the median times from Door-to-ECG (D2E) decreased significantly (159 min [IQR 83–340] to 29 min [IQR: 15–63], *p*-value = 0.001), as did the median time from ECG-to-STEMI team trigger (E2T) (31 min [IQR: 21–44] to 16 min [IQR: 6–40], *p*-value = 0.001) (Table 2). Additionally, the number of patients who underwent ECG within 12 h of symptom onset increased significantly (119 [41.4%] vs. 523 [87.5%], *p*-value = .001).

**Table 2:** Comparison of cardiac event response metrics between 2019–2020 and 2009–2010 cohorts.

Key Performance Metrics	2019–2020 (*n* = 598)	2009–2010 (*n* = 287)	*p*-value	Missing data^a^
Time from pain to first medical contact, median (IQR), min	122 [50–265]	180 [90–473]	0.003	82 (13.7)
Time from Door to ECG time, median (IQR), min	29 [15–63]	159 [83–340]	<0.001	49 (8.2)
Time from ECG to STEMI team trigger, median (IQR), min	16 [6–40]	31 [2–44]	<0.001	22 (3.6)
Door to fibrinolysis time, median (IQR), min	141 [92–202]	–^b^	–	13/209 (6.2)
Door to balloon time for primary PCI, median (IQR), min	240 [173–351]	–^b^	–	33/222 (14.8)
Any reperfusion	431 (72.0)	90 (31.3)	<0.001	–
Fibrinolytic use	209 (34.9)	59 (20.6)	<0.001	
Rescue PCI	24/209 (11.4)	–^b^		
Primary PCI	222 (37.1)	31 (10.8)	<0.001	
Coronary angiography	372 (62.2)	–^b^	–	49 (8.2)
No reperfusion therapy reason is symptom >12 h	61/167 (36.5)	129/197 (65.4)	<0.001	–

Data are presented as the number (percentage) of participants unless otherwise indicated.

^a^ Missing data is referent for 2019–2020 cohort.

^b^ Blank cells represent data not available between 2009 and 2010. Electrocardiogram (ECG); ST-segment elevation myocardial infarction (STEMI); Percutaneous Coronary Intervention (PCI); Not applicable (–).

There was a significant increase in overall reperfusion use, rising from 90 cases (31%) in the pre-intervention period to 431 cases (73%) in the post-intervention period (*p* = 0.001). This increase included isolated primary PCI, which rose from 31 cases (10.8%) to 222 cases (37.1%), and fibrinolytic use, which increased from 59 cases (20.6%) to 209 cases (34.9%) (both with *p* = 0.001) (Table 2). It is important to note that while door-to-needle and door-to-balloon times, as well as in-hospital mortality, still exceed international guidelines. Unfortunately, comparative data for these specific metrics from the 2009-2010 period are not available. The observed in-hospital mortality rate was 15%, with an average GRACE score of 109 (SD: 27).

## 4 Discussion

This study revealed a significant increase in the proportion of STEMI cases receiving reperfusion therapy, despite the expansion to a larger area and population assisted and the unchanged number of available hubs. This finding indicates the importance of optimizing protocols and efficiently dispatching patients within the critical treatment window.

In comparison to other regional registries, there is noticeable variability in the proportion of patients receiving reperfusion therapy: 90.1% in the Tamil Nadu STEMI registry (16), 92% in the Supra Network (17), 45% in the Veneto's region (18), and 37.2% in the JAC Registry (19). In Małopolska Registry, the rate varied from 65% to 89% depending on the region reported (20). The multinational LATIN group reported a 46% reperfusion rate among those diagnosed, with only 1.5% receiving fibrinolysis. This discrepancy suggests the need to overcome barriers to ensure broader access and utilization of reperfusion therapies (21, 22). These challenges may encompass logistical issues, limited resources, delays in patient transport, and variations in healthcare infrastructure and practices across different regions. There were significant reductions in all assistance times in Salvador's STEMI network. A median P2FMC time of 122 min was observed, which is lower than that reported by the Tamil Nadu registry, a region with similar social conditions (16), but it remains high when compared to standards from developed countries (23). Efforts to reduce these delays, which can subsequently decrease related complications and mortality rates, must prioritize public awareness on symptom recognition and the urgency of immediate medical care (24). However, specific measures to address this factor are lacking in the studied network. The reduction in the D2E and E2T times represents a significant improvement in the prompt identification of STEMI suspected cases by health care professionals and may be attributed to the implementation of continuous educational activities. However, reaching the 10-minute target for D2E time remains a challenge.

Regarding reperfusion times, the need to reduce D2N time remains evident despite data limitations that hinder direct comparisons with previous periods. The current median of 140 min falls significantly short of the quality benchmark. This discrepancy is also reflected in other registries, such as LATIN and CAMI, which report average fibrinolysis times of 205 min and a median time of 220 min, respectively, highlighting real-world challenges in adhering to guideline recommendations (22, 25). Similarly, a regional registry from São Paulo city, Brazil, which only provides pharmacoinvasive therapy due to logistical constraints, reported minimal changes in D2N time over a decade. Their median D2N time was 70 [43–115] minutes. Both registries underscore the urgent need to shorten time to reperfusion strategies (26).

In this study, community-based emergency units served as the primary medical contact point, while EMS ambulances were responsible for the swift dispatch of fibrinolytics or the transfer of patients to hubs. Nonetheless, there is ongoing debate about whether having EMS ambulances as the primary medical contact point could contribute to the reduction of reperfusion times. Such a strategy could prevent transferring patients to units lacking essential resources, such as PPCI, and has been stated as good practice for many studies (2, 27–29).

A notable 108% increase in diagnosed STEMI cases was recorded over the study period. National public data on the prevalence of STEMI in Brazil are limited; however, available data on AMI-related hospitalizations indicate an upward trend in the network's assisted area during the same timeframe (30). The number of cases rose from 1,450 to 3,355, representing a 131% increase. While these general AMI data provide valuable insights into the overall trends of myocardial infarction, they may not fully capture the specific dynamics of STEMI cases in Brazil.

The observed in-hospital mortality rate of 15% is alarming. A previous study from Salvador's STEMI Network, reported a comparable 30-day mortality rate of 15% between the years 2011 to 2013, along with an average GRACE score of 145 ± 34 (31). It is essential to recognize that mortality rates can vary significantly across different cities and countries. For instance, the registry of Veneto in Italy, that operates in a similar model, reported a 12.2% in-hospital mortality rate (18). The Małopolska registry (20) reported a 19.3% in-hospital mortality, and in contrast, the LATIN registry evidenced 5.1% mortality rate (22), the Tamil Nadu and Supra STEMI registry 5.6% (16, 17), and the JAC registry 7.5% (19).

### 4.1 Limitations

The primary limitation of this study is the lack of continuous information since the implementation of the STEMI network. This gap led to incomplete data storage, preventing comprehensive tracking of important time metrics and mortality rates over the entire duration of the network. This interruption in data collection can be attributed to the initial absence of professionals directly employed by the STEMI network. Additionally, certain benchmarks in STEMI evaluation, such as total ischemic time and Door-In-Door-Out time, were not reported in this study, further contributing to data gaps. Despite these limitations, this study provides valuable insights into the long-term performance of a STEMI network in a low- and middle-income country setting and highlights areas for improvement in future evaluations.

### 4.2 Future perspectives

New challenges, ongoing challenges, and achievements in the post-intervention period were summarized in Table 3. There is a pressing need for effective initiatives to educate patients on recognizing acute coronary syndrome symptoms and the urgency of immediate medical intervention, which could reduce P2FMC times. Another important issue is the shortage of beds in coronary care units, which affects the delivery of timely specialized care.

**Table 3:** A decade of progress and ongoing challenges in salvador's STEMI network.

Challenges	Infrastructure	Professional training and population health education	Management and coordination of the health system
Persistent	Low level of health system informatization with a lack of technology for specific record	Education for patients’ recognition of ACS symptoms	STEMI care times above recommended by the guidelines
	Insufficient availability of hospital beds in coronary care units/hubs		
Accessed	Varied level and capacity of health facilities	Absence of clear leadership and predefined roles in some spokes	Lack of standardization of STEMI care protocols
	Increased assisted network		Limited sharing of information with private system units
	Insufficient support team	Periodic training for spokes’ staff	Extended response time (more than 60 s)
New	Provide the availability of fibrinolytics on a large scale among the spokes	High turnover of health professionals in the spokes	Offer individualized performance feedback to spokes

ST-segment–elevation myocardial infarction (STEMI); Acute coronary syndrome (ACS); Emergency medical service (EMS).

Regarding the network's spokes, a high turnover of frontline professionals impairs the consistency and standardization of STEMI care. Additionally, the limited availability of electronic medical records and digital ECGs may cause significant communication delays. Furthermore, the high cost and lack of staff experience in using fibrinolytics hampers the widespread availability of these medications in the network. However, the decentralization of healthcare during the COVID-19 pandemic has led to greater availability of these medications in the network.

## 5 Conclusion

The strategies adopted by Salvador's STEMI network on this decade-long analysis were associated with significant improvements in the reperfusion delivery rate and in door-to-ECG and ECG-to-Network trigger times. However, despite its efforts, the mortality rate remains high.

## Data Availability

The raw data supporting the conclusions of this article will be made available by the authors, without undue reservation.

## References

[1] Vaduganathan M Mensah GA Turco JV Fuster V Roth GA. The global burden of cardiovascular diseases and risk: a compass for future health. J Am Coll Cardiol. (2022) 80(25):2361–71. 10.1016/j.jacc.2022.11.005 36368511

[2] Tubaro M. An organized system of emergency care for patients with myocardial infarction: a reality? Future Cardiol. (2010) 6(4):483–9. 10.2217/fca.10.25 20608821

[3] Danchin N. Systems of care for ST-segment elevation myocardial infarction: impact of different models on clinical outcomes. JACC Cardiovasc Interv. (2009) 2(10):901–8. 10.1016/j.jcin.2009.05.025 19850247

[4] Astarcioglu MA Sen T Kilit C Durmus HI Gozubuyuk G Kalcik M Time-to-reperfusion in STEMI undergoing interhospital transfer using smartphone and WhatsApp messenger. Am J Emerg Med. (2015) 33(10):1382–4. 10.1016/j.ajem.2015.07.029 26299691

[5] Huber K Gersh BJ Goldstein P Granger CB Armstrong PW. The organization, function, and outcomes of ST-elevation myocardial infarction networks worldwide: current state, unmet needs and future directions. Eur Heart J. (2014) 35(23):1526–32. 10.1093/eurheartj/ehu125 24742888

[6] Ornato JP. Accelerating time to reperfusion in acute myocardial infarction: prehospital and emergency department strategies, systems of care, and pharmacologic interventions. Rev Cardiovasc Med. (2006) 7(Suppl 4):S49–60. 17224891

[7] Chandrashekhar Y Alexander T Mullasari A Kumbhani DJ Alam S Alexanderson E Resource and infrastructure-appropriate management of ST-segment elevation myocardial infarction in low- and middle-income countries. Circulation. (2020) 141(24):2004–25. 10.1161/CIRCULATIONAHA.119.041297 32539609

[8] Solla DJF de Mattos Paiva Filho I Delisle JE Braga AA de Moura JB de Moraes X Integrated regional networks for ST-segment–elevation myocardial infarction care in developing countries. Circ Cardiovasc Qual Outcomes. (2013) 6(1):9–17. 10.1161/CIRCOUTCOMES.112.967505 23233748

[9] Ogrinc G Davies L Goodman D Batalden P Davidoff F Stevens D. SQUIRE 2.0 (standards for QUality improvement reporting excellence): revised publication guidelines from a detailed consensus process. BMJ Qual Saf. (2016) 25(12):986–92. 10.1136/bmjqs-2015-004411 26369893 PMC5256233

[10] Malvestio MAA Sousa RMCD. Força de trabalho do SAMU 192 no Brasil: Composição, capacidade operacional e procedimentos atribuídos. (2024). Available online at: https://preprints.scielo.org/index.php/scielo/preprint/view/4911/version/8849 (cited July 4, 2024)

[11] Machado CV Salvador FGF O’Dwyer G. Serviço de atendimento móvel de urgência: análise da política brasileira. Rev Saúde Pública. (2011) 45:519–28. 10.1590/S0034-89102011005000022 21503554

[12] Piegas LS Timerman A Feitosa GS Nicolau JC Mattos LAP Andrade MD V diretriz da sociedade brasileira de cardiologia sobre tratamento do infarto agudo do miocárdio com supradesnível do segmento ST. Arq Bras Cardiol. (2015) 105:1–121. 10.5935/abc.20150107 26375058

[13] Ibanez B James S Agewall S Antunes MJ Bucciarelli-Ducci C Bueno H 2017 ESC guidelines for the management of acute myocardial infarction in patients presenting with ST-segment elevation: the task force for the management of acute myocardial infarction in patients presenting with ST-segment elevation of the European Society of Cardiology (ESC). Eur Heart J. (2018) 39(2):119–77. 10.1093/eurheartj/ehx393 28886621

[14] QGIS Development Team. QGIS Geographical Information System. Open Source Geospatial Foundation Project (2009). Available online at: http://qgis.osgeo.org

[15] Portal de Dados Abertos. Available online at: https://dados.gov.br/dados/organizacoes/visualizar/instituto-brasileiro-de-geografia-e-estatistica-ibge (cited July 4, 2024).

[16] Alexander T Mullasari AS Joseph G Kannan K Veerasekar G Victor SM A system of care for patients with ST-segment elevation myocardial infarction in India: the Tamil nadu–ST-segment elevation myocardial infarction program. JAMA Cardiol. (2017) 2(5):498–505. 10.1001/jamacardio.2016.5977 28273293 PMC5814984

[17] Teixeira AB Zancaner LF Ribeiro FF de F Pintyá JP Schmidt A Maciel BC Otimização da terapia de reperfusão no infarto agudo do miocárdio com supradesnível do segmento ST por meio de telemedicina baseada no WhatsApp®. Arq Bras Cardiol. (2022) 118(3):556–64. 10.36660/abc.20201243 35137785 PMC8959040

[18] Saia M Mantoan D Fonzo M Bertoncello C Soattin M Sperotto M Impact of the regional network for AMI in the management of STEMI on care processes, outcomes and health inequities in the veneto region, Italy. Int J Environ Res Public Health. (2018) 15(9):1980. 10.3390/ijerph15091980 30208613 PMC6163929

[19] Dharma S Andriantoro H Dakota I Purnawan I Pratama V Isnanijah H Organisation of reperfusion therapy for STEMI in a developing country. Open Heart. (2015) 2(1):e000240. 10.1136/openhrt-2015-000240 26019883 PMC4442233

[20] Dudek D Siudak Z Dziewierz A Rakowski T Mielecki W Brzeziński M Original article local hospital networks for STEMI treatment for a population of half a million inhabitants increase the use of invasive treatment of acute coronary syndromes to the European recommended level. The Małopolska registry of acute coronary syndromes 2005–2006. Pol Heart J Kardiologia Pol. (2008) 66(5):489–97. 18537056

[21] Mehta S Aboushi H Campos C Botelho R Fernandez F Rodriguez D Impact of a telemedicine-guided, population-based, STEMI network on reperfusion strategy, efficiency, and outcomes. AsiaIntervention. (2021) 7(1):18–26. 10.4244/AIJ-D-18-00047 34912998 PMC8657047

[22] Mehta S Grines CL Botelho R Fernandez F Cade J Dusilek C STEMI telemedicine for 100 million lives. Catheter Cardiovasc Interv Off J Soc Card Angiogr Interv. (2021) 98(6):1066–71. 10.1002/ccd.29896 34347365

[23] Di Pasquale G. The avoidable delay in the care of STEMI patients is still *a priori*ty issue. Int J Cardiol Heart Vasc. (2022) 39:101011. 10.1016/j.ijcha.2022.101011 35402689 PMC8984632

[24] Balbaa A ElGuindy A Pericak D Natarajan MK Schwalm J. Before the door: comparing factors affecting symptom onset to first medical contact for STEMI patients between a high and low-middle income country. IJC Heart Vasc. (2022) 39:100978. 10.1016/j.ijcha.2022.100978 35402688 PMC8984626

[25] Wu C Li L Wang S Zeng J Yang J Xu H Fibrinolytic therapy use for ST-segment elevation myocardial infarction and long-term outcomes in China: 2-year results from the China acute myocardial infarction registry. BMC Cardiovasc Disord. (2023) 23:103. 10.1186/s12872-023-03105-1 36814182 PMC9948459

[26] De Marqui Moraes PI Galhardo A Barbosa AHP de Sousa JMA Alves CMR Bianco HT Metrics of care and cardiovascular outcomes in patients with ST-elevation myocardial infarction treated with pharmacoinvasive strategy: a decade-long network in a populous city in Brazil. BMC Cardiovasc Disord. (2023) 23:300. 10.1186/s12872-023-03340-6 37322425 PMC10268408

[27] Kakou-Guikahue M N’Guetta R Anzouan-Kacou JB Kramoh E N’Dori R Ba SA Optimizing the management of acute coronary syndromes in Sub-saharan Africa: a statement from the AFRICARDIO 2015 consensus team. Arch Cardiovasc Dis. (2016) 109(6–7):376–83. 10.1016/j.acvd.2015.12.005 27020513

[28] Granger CB Bates ER Jollis JG Antman EM Nichol G O’Connor RE Improving care of STEMI in the United States 2008 to 2012. J Am Heart Assoc. (2019) 8(1):e008096. 10.1161/JAHA.118.008096 30596310 PMC6405711

[29] Rokos IC French WJ Koenig WJ Stratton SJ Nighswonger B Strunk B Integration of pre-hospital electrocardiograms and ST-elevation myocardial infarction receiving center (SRC) networks: impact on door-to-balloon times across 10 independent regions. JACC Cardiovasc Interv. (2009) 2(4):339–46. 10.1016/j.jcin.2008.11.013 19463447

[30] Brasil M da S. DATASUS. Morbidade Hospitalar do SUS - por local de residência - Bahia. Available online at: http://tabnet.datasus.gov.br/cgi/tabcgi.exe?sih/cnv/nrba.def (cited July 23, 2023).

[31] Filgueiras Filho NM Feitosa Filho GS Solla DJF Argôlo FC Guimarães PO Paiva Filho I de M Implementation of a regional network for ST-segment–elevation myocardial infarction (STEMI) care and 30-day mortality in a low- to middle-income city in Brazil: findings from salvador’s STEMI registry (RESISST). J Am Heart Assoc. (2018) 7(14):e008624. 10.1161/JAHA.118.008624 29980522 PMC6064829

